# The association between naturally acquired IgG subclass specific antibodies to the PfRH5 invasion complex and protection from *Plasmodium falciparum* malaria

**DOI:** 10.1038/srep33094

**Published:** 2016-09-08

**Authors:** Rupert Weaver, Linda Reiling, Gaoqian Feng, Damien R. Drew, Ivo Mueller, Peter M. Siba, Takafumi Tsuboi, Jack S. Richards, Freya J. I. Fowkes, James G. Beeson

**Affiliations:** 1 Centre for Biomedical Research, Burnet Institute, Melbourne, Australia; 2 Department of Medicine, Royal Melbourne Hospital, University of Melbourne, Parkville, Australia; 3 Population Health and Immunity Division, Walter and Eliza Hall Institute of Medical Research, Parkville, Australia; 4 Department of Medical Biology, University of Melbourne, Melbourne, Victoria, Australia; 5 ISGlobal, Barcelona Centre for International Health Research (CRESIB), Hospital Clinic, University of Barcelona, Barcelona, Spain; 6 Papua New Guinea Institute for Medical Research, Goroka, Papua New Guinea; 7 Division of Malaria Research, Proteo-Science Center, Ehime University, Matsuyama, Japan; 8 Department of Microbiology and Central Clinical School, Monash University, Melbourne, Australia; 9 Centre for Epidemiology and Biostatistics, Melbourne School of Population and Global Health, University of Melbourne, Australia; 10 Department of Epidemiology and Preventive Medicine and Department of Infectious Diseases, Monash University, Melbourne, Australia

## Abstract

Understanding the targets and mechanisms of human immunity to malaria is important for advancing the development of highly efficacious vaccines and serological tools for malaria surveillance. The PfRH5 and PfRipr proteins form a complex on the surface of *P. falciparum* merozoites that is essential for invasion of erythrocytes and are vaccine candidates. We determined IgG subclass responses to these proteins among malaria-exposed individuals in Papua New Guinea and their association with protection from malaria in a longitudinal cohort of children. Cytophilic subclasses, IgG1 and IgG3, were predominant with limited IgG2 and IgG4, and IgG subclass-specific responses were higher in older children and those with active infection. High IgG3 to PfRH5 and PfRipr were significantly and strongly associated with reduced risk of malaria after adjusting for potential confounding factors, whereas associations for IgG1 responses were generally weaker and not statistically significant. Results further indicated that malaria exposure leads to the co-acquisition of IgG1 and IgG3 to PfRH5 and PfRipr, as well as to other PfRH invasion ligands, PfRH2 and PfRH4. These findings suggest that IgG3 responses to PfRH5 and PfRipr may play a significant role in mediating naturally-acquired immunity and support their potential as vaccine candidates and their use as antibody biomarkers of immunity.

*Plasmodium falciparum* malaria is a leading cause of morbidity and mortality globally, particularly in young children^1^. The development of an effective vaccine would provide a powerful tool for malaria control and elimination^2^,^3^. A detailed knowledge of human immunity to malaria, especially identifying key targets and effector mechanisms that mediate protection, is needed to advance vaccine development. In malaria endemic areas individuals acquire immunity to symptomatic infection after repeated exposure, supporting the rationale for the development of a *P. falciparum* vaccine that is capable of generating protective immunity against malaria^3^,^4^,^5^,^6^,^7^,^8^. Acquired immunity largely targets blood-stage antigens and is proposed to function by limiting parasite replication, thereby preventing the development of high-density parasitemia and symptomatic malaria^3^,^9^. Antibodies to *P. falciparum* blood stage antigens have been shown to be an important part of this protective immune response^3^,^4^,^8^,^10^,^11^,^12^,^13^,^14^,^15^. However, the principal targets and precise mechanisms of protective immune responses remain poorly understood.

A promising emerging vaccine candidate is *P. falciparum* reticulocyte binding-like homologue protein (PfRH5)^16^,^17^ which has recently been reported to elicit protective antibody responses in a non-human primate vaccine trial^18^. The PfRH family of invasion ligands is expressed by *P. falciparum* merozoites and includes PfRH1, PfRH2a, PfRH2b, PfRH4 and PfRH5. While all five members of this family play important roles in merozoite invasion, only PfRH5 appears essential to blood stage replication of *P. falciparum in vitro*^13^,^19^,^20^,^21^. The PfRH5 protein is located in the rhoptries, but is secreted onto the surface of merozoites prior to RBC invasion^13^. After its release, PfRH5 forms a complex on the merozoite surface with PfRipr (*P. falciparum* PfRH5 interacting protein)^22^ and CyRPA (Cysteine-rich protective antigen)^23^,^24^. The exact mechanism by which this complex is tethered to the merozoite surface remains the subject of some debate. It has been proposed that CyRPA is GPI-anchored to the merozoite surface^24^, whereas others have suggested that it is secreted^23^ leaving open the possibility that the PfRH5 complex attaches to the merozoite surface through an additional or alternate component. PfRH5 binds to basigin on the surface of red blood cells (RBC)^25^. Antibodies to all three components of the complex can inhibit parasite invasion^13^,^22^,^24^.

Individuals naturally exposed to *P. falciparum* in malaria endemic regions of Africa and Papua New Guinea have been shown to develop anti-PfRH5 antibodies^5^,^26^,^27^, and antibodies to PfRH5 were associated with protection from symptomatic malaria in malaria-endemic regions of Papua New Guinea^5^, and Mali^27^. Antibodies to PfRipr have also been associated with protection from malaria in children^5^. Affinity-purified antibodies to PfRH5 from naturally-exposed individuals were shown to inhibit *P. falciparum* growth *in vitro*^26^,^27^. These findings suggest that PfRH5 complex is an important target of immunity. However the nature of the IgG subclass response to PfRh5 and PfRipr and how this relates to acquired protective immunity has not been established. The aims of the present study were to define the nature of IgG subclass responses to PfRH5 and PfRipr, and determine the associations between subclass-specific responses and the prospective risk of malaria. We tested the hypothesis that the response to PfRH5 and Pfripr is restricted to certain IgG subclasses, and that specific IgG subclass responses may be more strongly associated with protection. Furthermore, we aimed to assess the potential influence of host age and *P. falciparum* infection on acquisition of these antibodies and their relationship to the acquisition of antibodies to other PfRH invasion ligands.

## Methods

### Study population

The study population and details of the cohort study, conducted between June and December 2004, have been described previously^28^. Briefly, plasma samples were obtained from a treatment to re-infection study of 206 school children from Mugil and Megiar, Madang Province, Papua New Guinea. The children were aged between 5–14 years with a median age of 9.3 years. Blood samples were taken at enrolment (baseline). Following enrolment, children were treated with artesunate to clear infection and then actively monitored fortnightly for 6 months for re-infection and symptomatic malaria episodes. Re-infection and treatment failure were distinguished by MSP2 genotyping. Plasma samples taken at enrolment were used for analysis by ELISA. Negative controls were taken from malaria-naïve Australian residents and positive controls were from malaria-exposed adults from PNG and were used to standardise assays and account for variation between plates.

Ethics approval was obtained from the Medical Research Advisory Committee, PNG, the Walter and Eliza Hall Institute of Medical Research Ethics Committee, and The Alfred Human Research Ethics Committee and the study was conducted in accordance with the protocol and relevant guidelines. Written informed consent was obtained from all study participants or their guardians (for children).

### Recombinant proteins

Recombinant PfRH5 and PfRipr were expressed in a wheat germ cell-free expression system (WGCF)^29^. PfRH5 comprised the full ectodomain (amino acid residues 25–526). PfRipr comprised amino acids 238–368^5^. Both proteins were assessed for quality and purity by SDS-PAGE and Western blot (reduced and non-reduced), as described^5^. Previous studies have demonstrated that antibodies measured by ELISA to recombinant PfRH5 and PfRipr are specific to malaria-exposed individuals, and the recombinant PfRH5 was shown to be functionally active, binding to the RBC surface, and able to generate inhibitory antibodies when used in immunizations^30^.

### Antibodies by ELISA

ELISA (Enzyme Linked Immunosorbent Assays) were performed in 96-well plates (NUNC, Denmark) using established methods described elsewhere^6^. All proteins, sera and reagents were used at 50 μl/well. Proteins were coated onto plates in PBS solution at a concentration of 2 μg/ml and incubated overnight at 4 °C. Plates were blocked with 200 μl of 10% skim milk in PBS/0.05% Tween for 2 hours. Plasma samples were tested at 1:250 in 5% milk in PBS/0.05% Tween solution for 2 hours at room temperature. Mouse anti-human IgG1 and 3 (Invitrogen life technologies, USA) were used at 1:500 to detect IgG subclass antibodies and incubated for 1 hour at room temperature. Tertiary antibody (goat anti-mouse HRP conjugated, Merck Millipore, USA) was added at 1:250 dilution for 1 hour at room temperature. ABTS liquid substrate system (Sigma-Aldrich, USA) was added to measure enzymatic activity. All wash steps were done with PBS/0.05% Tween. OD was measured at 405 nm. Plate-to-plate variation was standardized using positive controls on each plate and plasma from non-immune blood donors were used as negative controls. Seropositivity was defined as an OD above the highest value obtained from the selection of negative controls. All samples were tested in duplicate. Reagents and sera were tested in serial dilution to determine the optimum dilution for testing the cohort. Previous studies have shown the use of mid-point dilutions provide results that strongly correlate with end-point titrations; therefore one midpoint dilution was chosen^10^,^31^. Data for total IgG to PfRH5 and PfRipr were previously determined^5^. Pilot experiments were performed to ensure the specificity of subclass detection antibodies, which were validated against purified IgG subclass antibodies (Invitrogen). Optimization was carried out for all reagents, sera and incubation time to determine the optimal parameters to ensure a good separation between high, medium and low responders. Optimisation experiments included (i) testing secondary and tertiary antibodies across a range of dilutions in ELISA with each antigen, (ii) testing a selection of subject’s samples across a range of dilutions in ELISAs with antigen and IgG subclass, and (iii) testing recombinant antigens at a range of coating concentrations.

### Statistical analysis

Kruskal-Wallis and chi-squared tests were used to determine the association of continuous and categorical variables, respectively, with categorical variables. Correlations between antibody responses were assessed using Spearman’s rho (ρ). To assess the association between subclass responses and protection from symptomatic malaria, children were classified into three equal groups of high, medium and low responders, according to the OD of antibody responses; children who were negative for antibodies were included in the low responder group^6^. A symptomatic episode of *P. falciparum* malaria was defined as a fever (>37 °C) and a parasitemia >5000/μl^28^. Kaplan-Meier survival analyses were performed for time-to-first episode of symptomatic *P. falciparum* malaria, with subjects stratified as high, medium or low responders, and a log rank test was performed to compare response tertiles. Cox proportional hazard models were used to determine the association between antibody response categories and risk of symptomatic *P. falciparum* malaria adjusting for potential confounders^6^. A range of demographic, clinical and biological variables were assessed as potential confounders of associations between antibodies and malaria outcomes. Only host age and location of residence were identified as being significantly associated with antibodies and malaria outcomes^6^,^28^. Therefore, these two variables were included in the adjusted analyses. Other variables (consider *a priori*), such as parasitemia at enrolment, and red blood cell genetic polymorphisms, were not significantly associated with the outcome of malaria. Age was used as binary variable (≤9 years, >9 years), as previous studies indicated that this stratification was the most informative approach for assessing the effect of age^6^,^28^. Of note, parasitemia status at enrolment and red blood cell polymorphisms were not associated with malaria outcomes^28^. IgG1 response groups showed non-proportional hazards; therefore an interaction term between antibody level and time was included in the model (using three time categories t = 0–100, t = 100–150 and t > 150 days since study commencement)^7^.

## Results

### Subclass response to PfRH5 and PfRipr

**Figure 1:**
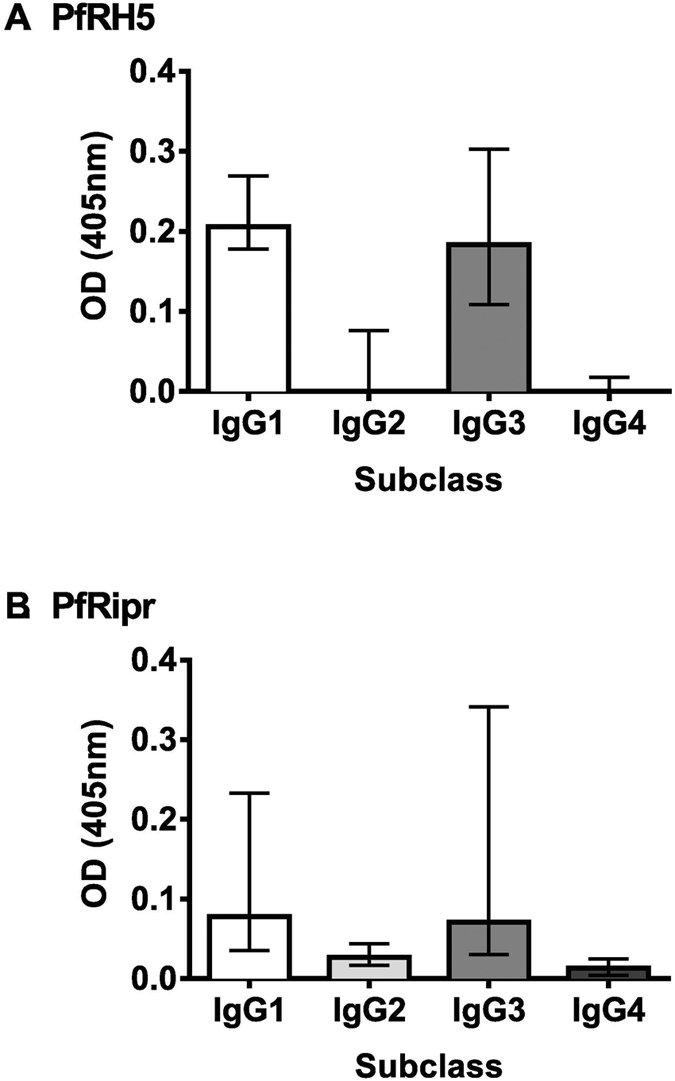
Reactivity of IgG subclasses to PfRH5 and PfRipr in PNG adults. Optical density (OD) values obtained for a selection of sera from malaria exposed PNG adults (n = 24) for (**A**) PfRH5 and (**B**) PfRipr specific IgG subclass reactivity. Bars represent median value. Error bars represent interquartile range.

To determine the predominant IgG subclass response to PfRH5 and PfRipr, a selection of malaria exposed PNG adults (n = 24) who were resident in Madang Province were tested. IgG1 and IgG3 were the predominant subclass response to both antigens (seroprevalence for PfRH5 IgG1: 91.0%, IgG3: 86%; PfRipr IgG1: 63%, IgG3: 59%; OD levels shown in Fig. 1). The predominance of IgG1 and IgG3 to these antigens is similar to IgG subclass responses to other merozoite antigens, including other PfRH family members PfRH2 and PfRH4^7^,^12^. Very few individuals were classified as seropositive for IgG4 to PfRH5 (4.5%) or PfRipr (4.5%). There was also a low seroprevalence for IgG2 to PfRipr (13%), but a higher proportion were classified as seropositive for IgG2 to PfRH5 (34.8%). However, it should be noted that levels of IgG2 reactivity were generally very low (Fig. 1), even though some samples were classified as seropositive. Given the limited reactivity of IgG2 and IgG4 observed, only IgG1 and IgG3 responses to PfRH5 and PfRipr were determined for the main study.

In the children’s cohort, IgG1 and IgG3 to PfRH5 and PfRipr had higher median ODs compared to malaria-naïve negative controls indicating the specificity of *P. falciparum* antibody responses (p < 0.001). IgG3 was the predominant subclass response against both antigens with a higher seroprevalence compared to IgG1 (p < 0.001) (PfRH5 seroprevalence: IgG1 32%, IgG3 57.3%; PfRipr seroprevalence: IgG1 47%, IgG3 62.1%).

### Association of IgG subclass responses to PfRH5 and PfRipr with age and concurrent parasitaemia

**Figure 2:**
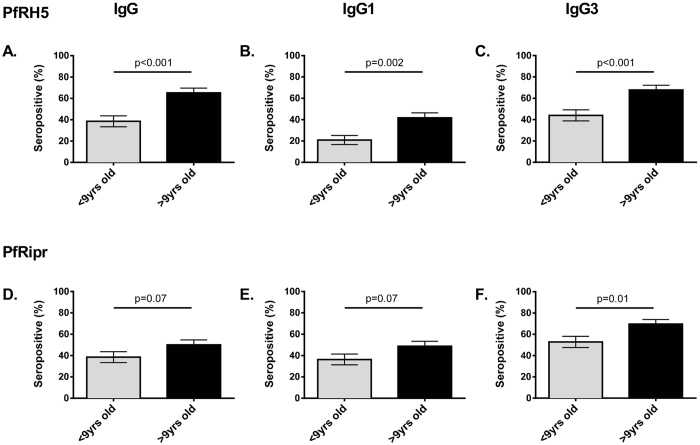
Seroprevalence of total IgG and subclass response to PfRH5 and PfRipr by age. IgG seroprevalence to PfRH5 and PfRipr recombinant proteins measured by ELISA. Results are shown as percentage seropositive.. Error bars indicate standard error of proportion (SE). Results are shown by Age (<9 or >9 years of age). Top row: PfRH5. Bottom row: PfRipr. Results are shown for (**A**)/(**D**) Total IgG, (**B**)/(**E**). IgG1 and (**C**)/(**F**) IgG3 left to right. P values indicate chi square test.

To investigate the relationships between age and IgG subclasses responses (IgG1 and IgG3) to PfRH5 and PfRipr, seroprevalence was compared between younger and older children (Fig. 2). The seroprevalence of total IgG to PfRH5 and PfRipr was significantly higher in older children compared to younger children (PfRH5, >9 yrs: 65.2%, <9 yrs: 38.5%, p < 0.001; PfRipr >9 yrs: 50.0%, <9 yrs: 38.5%, p = 0.07). Higher seroprevalence of IgG1 and IgG3 subclass responses were also found in older compared to younger children for both PfRH5 and PfRipr (PfRH5 IgG1: >9 yrs: 41.7%, <9 yrs: 20.9%, p = 0.002; IgG3: >9 yrs: 67.8%, <9 yrs: 44.0%, p < 0.001; PfRipr, IgG1, >9 yrs: 48.7%, <9 yrs: 36.3%, p = 0.07; IgG3 >9 yrs: 69.6%, <9 yrs: 52.8%, p = 0.01) (Fig. 2).

**Figure 3:**
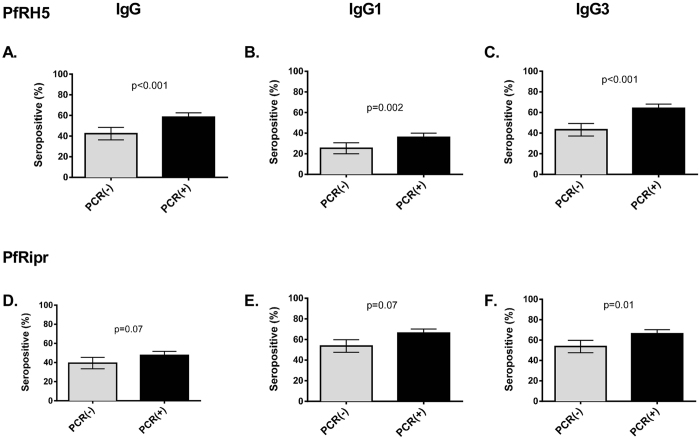
Seroprevalence of total IgG and subclass response to PfRH5 and PfRipr by concurrent parasitemia measured by PCR. Results are shown as percentage seropositive, as determined by ELISA. Error bars indicate standard error of proportion (SE). Results are shown by parasitemic status at enrolment (PCR(−) or PCR(+)). Top row: PfRH5 Bottom row: PfRipr. Results are shown for (**A**)/(**D**) Total IgG, (**B**)/(**E**) IgG1 and (**C**)/(**F**) IgG3 left to right. P values indicate chi square test.

Previous studies have shown individuals with active *P. falciparum* infection generally have higher levels of antibodies to merozoite antigens^4^,^6^,^32^. In our cohort, IgG3 and IgG1 responses to PfRH5 were significantly higher in PCR positive compared to PCR negative individuals. The prevalences of IgG3 responses were 43.3 and 64% for PCR(−) and PCR(+), respectively, (p = <0.001); prevelances of IgG1 responses were 25.4% and 36.0% (p = 0.002). PfRipr-specific IgG3 seroprevalence was higher in PCR positive compared to PCR negative individuals (PCR(−): 53.7%, PCR(+): 66.2%, p = 0.01), but the difference was less marked for IgG1 responses (PCR(−): 53.7%, PCR(+): 66.2%, p = 0.07) (Fig. 3).

### Relationships between antibody responses

Responses to PfRH5 were significantly positively correlated with each other (Table 1). Total IgG to PfRH5 was more strongly correlated with the predominant response of IgG3 than with IgG1 (IgG3: ρ = 0.76, IgG1: ρ = 0.58, p < 0.001). The two subclass responses to PfRH5 were also moderately correlated with one another (ρ = 0.49, p < 0.001). Total IgG to PfRipr was more strongly correlated with the major response of IgG3 compared to IgG1 (IgG3: ρ = 0.79 p < 0.001, IgG1: ρ = 0.69 p < 0.001). The two subclasses responses were also moderately correlated with one another (ρ = 0.53, p < 0.001). There was a very strong correlation between total IgG responses to both antigens (ρ = 0.82, p < 0.001). The predominant IgG3 responses to each antigen were also strongly correlated (ρ = 0.68, p < 0.001).

To investigate the co-acquisition of antibodies to different antigens, the total IgG and subclass responses to PfRH5 and PfRipr were compared to previously published data on IgG responses to other PfRH family proteins; PfRH2 (construct PfRH2-2030^7^) and PfRH4 (construct PfRH4.9^12^). Total and subclass IgG levels to all PfRH proteins were significantly correlated with each other (ρ range: 0.15–0.82, p < 0.05) (Table 1). The predominant IgG3 responses to PfRH5 were significantly moderately correlated to PfRH4 IgG1 and IgG3 (ρ = 0.32 and 0.46, respectively, p < 0.001) and PfRH2 IgG1 and IgG3 (ρ = 0.40 and 0.53, respectively, p < 0.001). The major subclass response to PfRipr, IgG3, was more strongly correlated with IgG3 responses to PfRH2 (ρ = 0.51, p < 0.001) and PfRH4 (ρ = 0.45, p < 0.001). These findings suggest there is co-acquisition of antibodies to PfRH5, PfRipr and other PfRH ligands following exposure to *P. falciparum* infection.

### Associations between IgG and subclass responses and clinical malaria

**Figure 4:**
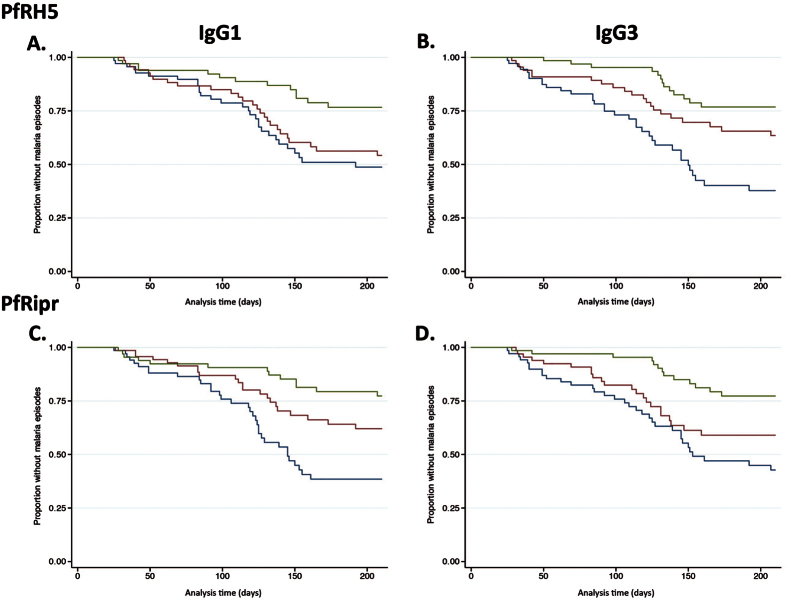
Risk of symptomatic *P. falciparum* episode during follow-up relative to IgG1 and IgG3 to PfRH5. Kaplan-Meier curves show the proportion of children that remained free of malaria episodes over time for IgG subclass responses against PfRH5 and PfRipr (**A**) IgG1 to PfRH5; (**B**) IgG3 to PfRH5; (**C**) IgG1 to PfRipr; (**D**) IgG3 to PfRH5. Antibody responses were divided into 3 equal response groups: high (green line), medium (red line), and low (blue line) antibody reactivity. Unadjusted data are shown. Wilcoxon log rank tests were carried out: (**A**) p = 0.004; (**B**) p < 0.001; (**C**) p < 0.001; (**D**) p < 0.001 Symptomatic *P. falciparum* infection was defined as fever (>37 °C) plus a parasite load of 5000 parasites/μl.

To investigate the association between antibody responses and protection from symptomatic *P. falciparum* malaria, the children were divided into three equal tertiles representing high, medium and low antibody levels for each subclass response and survival analyses were performed. In Kaplan-Meier survival analyses (unadjusted), there were significant differences between the three IgG subclass groups, with high responders having a longer time to first symptomatic *P. falciparum* episode than medium and low responders for both PfRH5 and PfRipr (PfRH5 IgG1: p = 0.004, IgG3: p < 0.001; PfRipr IgG1, IgG3 both p < 0.001) (Fig. 4).

After adjusting for the confounding effects of age and location of residence, individuals with high levels of total IgG to PfRH5 had a 65% lower risk of symptomatic *P. falciparum* malaria compared to individuals with low IgG levels, as reported previously^5^ (aHR 0.35, 95% CI: 0.12–0.96, p = 0.04) (Table 2). Similarly, individuals who were high responders for IgG3 to PfRH5 had a 65% decreased risk of symptomatic falciparum malaria compared to those who were classified as low responders, after adjustment for confounders; there was also a reduced risk of malaria for high IgG1 responders, but this did not reach statistical significance (IgG3: aHR 0.35, CI: 0.17–0.71, p = 0.03; and IgG1 aHR 0.46, CI: 0.16–1.29, p = 0.14) (Table 2). Total IgG against PfRipr was significantly associated with a 76% reduced risk of symptomatic *P. falciparum* malaria after adjusting for potential confounders, as previously reported^5^ (aHR: 0.24, 95% CI: 0.08–0.73, p = 0.01). As found for PfRH5 responses, high levels of IgG3 to PfRipr were strongly and significantly associated with protection from symptomatic *P. falciparum* malaria after adjusting for confounders (HvL responders aHR 0.36, 95% CI: 0.018-0.72, p = 0.004). Associations were weaker for IgG1 to PfRipr, and did not reach statistical significance after adjusting for confounders (aHR: 0.48 95% CI 0.19–1.26, p = 0.14).

## Discussion

In this study we found that cytophilic antibodies IgG1 and IgG3 were the predominant subclass responses to PfRH5 and PfRipr, which are present as an invasion complex on the merozoite surface. Interestingly, IgG3 responses were most strongly associated with protection from malaria in a longitudinal cohort study of children, whereas protective associations for IgG1-specific responses were weaker and did not reach statistical significance after adjusting for confounding variables. These findings provide further support for PfRH5 and PfRipr as malaria vaccine candidates and suggest that the IgG3 response is more important in mediating protection.

Our initial studies in a selection of adults indicated that IgG1 and IgG3 were the predominant responses to PfRH5 and PfRipr, with little IgG2 and IgG4 reactivity. In the larger children’s cohort, both IgG1 and IgG3 were prevalent, but IgG3 responses were significantly more common for both antigens. A prior study in Senegal found that IgG1 responses were more prevalent than IgG3 among adults (69% vs. 31% respectively), although the sample size in that study was small (n = 19)^27^. Previous studies suggest that the nature of the subclass response appears to be influenced by intrinsic properties of the antigen, the level of exposure to *P. falciparum* among individuals, and host factors^4^,^6^,^10^. We found that IgG subclass responses were somewhat higher among older children, consistent with acquisition of antibodies with increasing exposure to malaria^4^,^7^,^6^. Age is a measure of the broad level of exposure in coastal areas of PNG, which has year-round malaria transmission^28^. However, age may also be associated with changes in the physiological or immunological development; Baird *et. al*^33^. suggested that intrinsic changes in the immune response can contribute to protection. Therefore, our analyses were adjusted for age to account for the potential confounding influence of age^6^. IgG1 and IgG3 levels to both antigens tended to be higher in children with PCR-detectable *P. falciparum* infection compared to uninfected children. The overall trend of increased IgG subclass responses in those with concurrent parasitemia is consistent with other studies and reflect potential boosting of antibody responses with infection^6^,^34^,^35^.

A strength of this study is the longitudinal study design that enabled prospective examination of the association between antibodies and subsequent malaria episodes. We found that higher levels of IgG1 and IgG3 against PfRH5 and PfRipr at enrolment were associated with protection from subsequent symptomatic *P. falciparum* malaria during follow-up. There was evidence of a dose-response effect, as the risk of symptomatic *P. falciparum* malaria was significantly reduced in those with high compared to medium and low antibody levels. Protective associations were substantially stronger for IgG3 responses to PfRH5 and PfRipr than IgG1 responses, and only the associations between IgG3 and protection remained significant after adjustment for potential confounders. Interestingly, previous studies of IgG subclass responses to other members of the PfRH invasion ligand family found that IgG3 responses were strongly associated with protection from malaria^7^,^12^. Furthermore, IgG3 was also found to have the strongest protective association for the EBA invasion ligands, which act co-operatively with PfRH ligands, even when IgG1 was the predominant subclass produced^4^. High IgG3 responders to PfRH5 and PfRipr had a stronger association with protection compared to individuals with medium levels of IgG3 raising the possibility that there may be a threshold level of antibodies required to mediate effective protection. Protective associations for IgG3 responses to PfRH5 and PfRipr were strong in this cohort (HR values 0.26 and 0.29, respectively). The strength of these protective associations was stronger than we have previously reported in this cohort for IgG3 responses to some other antigens, such as MSP1 and MSP2, and similar to protective associations for IgG3 responses to EBA175, EBA140, and PfRH2^4^,^6^,^7^. In this study we evaluated antibodies only at enrolment. In future studies it would be valuable to evaluate PfRH5 and PfRipr antibody dynamics over time and evaluate the relationship between antibody levels prior to reinfection and subsequent risk of developing high density parasitemia. In our study we used recombinant proteins expressed using a wheat germ cell free expression system, and the antigens have been previously validated^5^. Others have found that IgG measured to recombinant PfRH5 expressed in E.coli and PfRipr expressed in baculovirus-infected Hi-5 cells among this same PNG cohort of children were associated with protection^36^, suggesting that the associations between PfRH5 or PfRipr responses and protection from malaria are robust.

Defining the nature of IgG subclass responses to PfRH5 and PfRipr, and their associations with protective immunity, is important because different subclasses have different immune effector functions^37^,^38^. As such, understanding the subclass response provides further insight into the possible biological function of antibodies and their potential role in protection. IgG1 and IgG3 are cytophilic and engage Fc-receptors to promote opsonic phagocytosis or killing by neutrophils; recent studies have reported that antibodies mediating opsonic phagocytosis of merozoites, or neutrophil-mediated killing, are associated with protection from malaria in children^15^,^39^. How IgG1 and IgG3 may differ in their opsonic phagocytosis functions for merozoites is not known, but differences between IgG1 and IgG3 have been reported in other systems^37^,^40^. IgG1 and IgG3 can also activate complement, and recent studies have suggested that human antibodies to merozoites interact with complement to inhibit invasion^41^. IgG3 is believed to be a more potent activator of complement^38^. Given the location of PfRH5 and PfRipr on the merozoite surface, their important role in invasion, and the finding that IgG3 is strongly associated with protection from malaria, future studies are needed to further evaluate the functional activity of antibodies to PfRH5 and PfRipr.

Total IgG, IgG1 and IgG3 to all PfRH family proteins were correlated, with the highest correlations observed with the predominant IgG subclass responses. IgG1 and IgG3 responses to PfRH5, or PfRipr, were found to be significantly correlated with each other. Furthermore, IgG1 and IgG3 to other PfRH family proteins, PfRH2 and PfRH4, were also correlated with PfRH5 and PfRipr responses. The predominant IgG subclass responses for each antigen were most strongly correlated. These findings indicate the co-acquisition of antibodies to multiple PfRH invasion ligands following exposure to *P. falciparum* infection, supporting a view that immunity to malaria is mediated by a repertoire of antibodies to multiple invasion ligands.

## Conclusion

This is the first study to examine the association of naturally-acquired IgG subclass responses to PfRH5 and PfRipr with protection from symptomatic *P. falciparum* malaria, finding that IgG3-specific responses are most strongly associated with immunity. This knowledge provides important insights into potential mechanisms mediating protection, and identifies possible antibody biomarkers of protective immunity that may have important applications for sero-surveillance in *P. falciparum* malaria endemic areas^42^. Further studies are needed to evaluate whether IgG3 responses to PfRH5 or PfRipr may be better biomarkers of protective immunity than total IgG responses to these antigens. The results from this study add to evidence supporting PfRH5 and PfRipr as potential candidates for inclusion in a multicomponent vaccine. Understanding the functional immunological significance between IgG1 and IgG3 responses to these antigens may be important for determining whether vaccines based on these antigens need to generate a particular IgG subclass response in order to maximize vaccine efficacy.

## Additional Information

**How to cite this article**: Weaver, R. *et al.* The association between naturally acquired IgG subclass specific antibodies to the PfRH5 invasion complex and protection from *Plasmodium falciparum* malaria. *Sci. Rep.* **6**, 33094; doi: 10.1038/srep33094 (2016).

## Figures and Tables

**Table 1:** Spearman’s ρ between IgG, IgG1 and IgG3 to PfRH family proteins: PfRH5; PfRipr; PfRH2; PfRH4.

		PfRh5			PfRipr			PfRh2			PfRh4		
		IgG	IgG1	IgG3	IgG	IgG1	IgG3	IgG	IgG1	IgG3	IgG	IgG1	IgG3
PfRh5	IgG	…	…	…	…	…	…	…	…	…	…	…	…
	IgG1	0.58**	…	…	…	…	…	…	…	…	…	…	…
	IgG3	0.76**	0.49**	…	…	…	…	…	…	…	…	…	…
PfRipr	IgG	0.82**	0.56**	0.76**	…	…	…	…	…	…	…	…	…
	IgG1	0.41**	0.46**	0.41**	0.69**	…	…	…	…	…	…	…	…
	IgG3	0.64**	0.52**	0.68**	0.79**	0.53**	…	…	…	…	…	…	…
PfRh2	IgG	0.36**	0.19*	0.41**	0.34**	0.27**	0.31**	…	…	…	…	…	…
	IgG1	0.35**	0.19**	0.40**	0.34**	0.29**	0.32**	0.88**	…	…	…	…	…
	IgG3	0.51**	0.23**	0.53**	0.45**	0.27**	0.51**	0.43**	0.46**	…	…	…	…
PfRh4	IgG	0.32**	0.15*	0.35**	0.3**	0.24**	0.28**	0.64**	0.69**	0.41**	…	…	…
	IgG1	0.32**	0.15*	0.32**	0.28**	0.22*	0.28**	0.64**	0.70**	0.42**	0.93**	…	…
	IgG3	0.44**	0.15*	0.46**	0.39**	0.35**	0.45**	0.35**	0.44**	0.55**	0.39**	0.39**	…

Spearman ρ are shown for total IgG and subclasses IgG1 and 3 to PfRH5; PfRipr; PfRH4; PfRH2 in 206 Papua New Guinean children. * p < 0.05, ** p < 0.001. PfRH2 construct was PfRH2.2030. PfRH4 construct was PfRH4.9.

**Table 2:** Association between antibodies and risk of clinical malaria.

Antigen	Antibody	Comparison	HR (95% CI)	p Value	aHR(95% CI)	p Value
PfRh5	IgG (Total)	MvL	0.46 (0.2–1.06)	0.07	0.53 (0.23–1.25)	0.15
	IgG (Total)	HvL	0.27 (0.1–0.72)	0.01	0.35 (0.12–0.96)	0.04
	IgG1	MvL	0.81 (0.37–1.79)	0.61	0.98 (0.44–2.183)	0.97
	IgG1	HvL	0.36 (0.13–.99)	0.05	0.46 (0.16–1.29)	0.14
	IgG3	MvL	0.49 (0.28–0.84)	0.01	0.54 (0.31–0.95)	0.03
	IgG3	HvL	0.26 (0.14–0.50)	0.01	0.35 (0.17–0.71)	0.03
PfRipr	IgG (Total)	MvL	0.41 (0.18–0.94)	0.04	0.51 (0.22–1.21)	0.13
	IgG (Total)	HvL	0.2 (0.07–0.6)	0.004	0.24 (0.08–0.73)	0.01
	IgG1	MvL	0.53 (0.23–1.22)	0.14	0.6 (0.26–1.38)	0.23
	IgG1	HvL	0.38 (0.15–0.99)	0.05	0.48 (0.19–1.26)	0.14
	IgG3	MvL	0.66 (0.38–1.13)	0.13	0.79 (0.45–1.38)	0.41
	IgG3	HvL	0.29 (0.15–0.55)	<0.001	0.36 (0.18–0.72)	0.004

Study participants were stratified into 3 equal groups according to low, medium or high levels of PfRH5-specific or PfRipr-specific antibodies antibodies. Hazard ratios were calculated comparing high versus low levels of antibodies (HvL) and medium versus low levels (MvL) of antibodies for the risk of symptomatic malaria over 6 months of follow-up; analysis was based on first episode only. Unadjusted hazard ratios (HR) and adjusted hazard ratios hazard ratios (aHR; adjusted for age and location of residence) with 95% confidence intervals [95% CI] were calculated. During the follow-up period, 80 children experienced at least one episode of clinical malaria.
